# Nosocomial oral myiasis in ICU patients: occurrence of three sequential cases

**DOI:** 10.3205/dgkh000259

**Published:** 2015-12-01

**Authors:** Hamed Ebrahimzadeh Leylabadlo, Hossein Samadi Kafil, Mohammad Aghazadeh, Teimour Hazratian

**Affiliations:** 1 Infectious Disease and Tropical Medicine Research Center, Tabriz University of Medical Sciences, Tabriz, Iran; 2 Drug Applied Research Center, Tabriz University of Medical Sciences, Tabriz, Iran; 3 Department of Parasitology, Faculty of Medical Sciences, Tabriz University of Medical Sciences, Tabriz, Iran

**Keywords:** nosocomial myiasis, oral, hospital, flies

## Abstract

Myiasis is the infestation of living vertebrates or humans tissues by dipterous larvae. The oral cavity is rarely affected by this infestation and the circumstances which can lead to oral myiasis include persistent mouth opening together with poor hygiene. Such infestations have been reported mainly in developing countries such as in Asia. Although rare, nosocomial myiasis must be noted carefully, especially in case of hospitalized patients. This report describes three cases of nosocomial oral myiasis in hospitalized patients in ICU (intensive care unit) in Tabriz, North West of Iran.

## Zusammenfassung

Bei der Myiasis handelt es sich um die Infestation von Fliegenmaden in lebenden Geweben von Vertebraten oder des Menschen. Die Mundhöhle wird selten von dieser Infestation betroffen. Die Umstände, die zu einem Befall führen können, beinhalten ein persistierendes Offenstehen des Mundes in Verbindung mit schlechter Hygiene. Berichte über derartige Infestationen stammen vor allem aus Entwicklungsländern z.B. in Asien. Trotz des seltenen Vorkommens muss eine nosokomiale Myiasis vor allem bei hospitalisierten Patienten sorgfältig beachtet werden. Beschrieben werden drei konsekutive nosokomiale Fälle von oraler Myiasis bei hospitalisierten Intensivtherapiepatienten in Tabriz, Nordwestiran.

## Introduction

Myiasis is a pathogenic condition found in live humans and animals caused by various species of dipteran larvae and the larvae may invade the mouth, nose, eye, lung, ear, anus, and vagina [1]. Oral myiasis is regarded as a type of wound myiasis and it was first described by Laurence in 1909 and occurs in humans mainly in the tropics [2]. Oral myiasis is associated with poor oral hygiene, but other factors can also play a role, such as alcoholism, senility, suppurating lesions, gingival diseases, trauma, paralysis or immobility, and mental debility [3], [4]. 

Nosocomial myiasis is generally considered to be a rare event, but its occurence in hospitals either in a highly urbanized or a developing country is unavoidable to some extent. Cases have been reported from several countries in the world [5], [6], [7]. This report describes three cases of nosocomial oral myiasis that occur in ICU patients in the general hospital in Iran.

## Case descriptions

### Case No. 1

A 81-year-old Iranian man with a history of hypertension (HTN) and hemodialysis presented to the emergency department with complains of shortness of breath with cough since 5 days ago. He was intubated on the same day upon admission due to respiratory distress and respiratory arrest occurred, cardiopulmonary resuscitation (CPR) was performed and the patient was sent to the ICU. Five days after hospitalization, eight fly larvae were discovered in the aspiration material of the oral cavity. The larvae were removed using sterile forceps and sent to the microbiology unit in 70% alcohol solution. 

### Case No. 2

A 47-year-old Iranian man was admitted to an ICU in Iran. The patient was diagnosed with hepatitis B and liver cirrhosis. Due to respiratory apnea, loss of consciousness and right upper quadrant (RUQ) pain, he was intubated and underwent mechanical ventilation (MV). He has been in ICU for 15 days and on the sixth day of hospitalization, about 20 maggots which showed spiral form and varied in size from 4 to 6 mm were discovered in the oral cavity. The maggots were removed and sent to the microbiology unit laboratory.

### Case No. 3

A 74-year-old Iranian woman with clinical history of asthma for 5 years, was admitted with dyspnea and chest pain symptoms to the emergency room (ER) of the general hospital. Emergency treatment was performed, including intubation. She was intubated 5 days prior to admission to ICU. Several hours after transfer to the ICU, the nurse reported approximately 8 larvae in the patient’s oral cavity and removed the larvae by combining manual extraction with tweezers and aspiration after having washed the infected area with water and povidone iodine. But three hours later, the nurse reported 4 maggots which were removed and then sent to the microbiology unit laboratory.

## Origin and identification of larva

**Figure 1:**
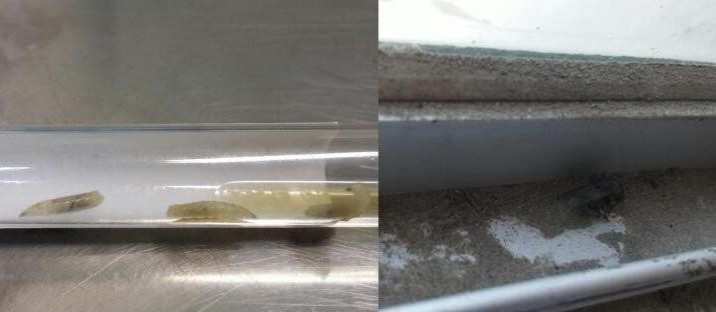
Macro photograph of the removed larvae and dead adult flies beside the window

These patients were classified as having nosocomial infestation. The cases occurred simultaneously in general ICU among three patients in late spring (June). The window meshes had been changed before the patients were hospitalized. Considering that it was the season for flies to bear their nits, some flies could have entered the ICU and bore their nits in the mouths of these three patients. Based on the morphological characteristics including size of the larva, spinous bands and the color in all the 3 cases, the larvae were identified as part of the Calliphoridae family (Figure 1 (Fig. 1)). In addition, through our inspection in the hospital, some dead adult flies were found beside the window (Figure 1 (Fig. 1)).

## Discussion

Nosocomial myiasis, which occurs in hospitalized patients, is a very infrequent phenomenon and oral myiasis is a rare process in developed countries, but can occur anywhere [8]. The risk factors for oral myiasis include suppurative lesions, trauma in the face, mouth-breathers and others conditions which commonly affects the anterior part of the mouth particularly the palate [9].

Nosocomial myiasis occurs worldwide in ICUs, but most of these rare cases will not be reported officially because of certain reasons [10]. In Iran country, there have been several case reports of myiasis in ICU patients [11], [12]. Recently the reported rates for nosocomial myiasis infections in Iran were about 4% and the proved mortality rate was 1.3% [13].

Our patients were intubated and bound to the mechanical ventilator with poor oral hygiene; conditions which combined predispose them to oral myiasis. We believe that the patients were lying with mouth half-open and this could explain the entrance of flies into the patient’s oral cavity and the laying of egg which hatched when the patients were in ICU.

Disease should be prevented by controlling the fly population and careful oral examination to identify less common diseases, especially in intubated unconscious patients. Education of medical and paramedical teams to sensitize for this problem should be performed. Also screening of the window meshes and other equipment in ICU is necessary.

## Notes

### Acknowledgement

The authors are thankful to the staff of the Department of Microbiology, Imam Reza Hospital for their collaboration.

### Competing interests

The authors declare that they have no competing interests.
